# Comparative Transcriptome Analysis of High- and Low-Growth Genotypes of *Eucalyptus urophylla* in Response to Long-Term Nitrogen Deficiency

**DOI:** 10.3390/genes15010060

**Published:** 2023-12-30

**Authors:** Xiaohui Yang, Fang Xu, Wen Pan, Weihua Zhang, Huanqin Liao, Baozhu Zhu, Bin Xu, Xinyu Chen, Huixiao Yang

**Affiliations:** 1 Guangdong Provincial Key Laboratory of Silviculture, Protection and Utilization, No. 233, Guangshan First Road, Guangzhou 510520, China; xiaohuiyang@sinogaf.cn (X.Y.); xufang@sinogaf.cn (F.X.); panwen@sinogaf.cn (W.P.); zwh523@sinogaf.cn (W.Z.); liaohuanqin@sinogaf.cn (H.L.); xubin@sinogaf.cn (B.X.); chenxinyu@sinogaf.cn (X.C.); 2 Guangdong Academy of Forestry, No. 233, Guangshan First Road, Guangzhou 510520, China

**Keywords:** nutrient starvation, comparative transcriptome analysis, stress resistance, *E. urophylla*

## Abstract

Nutrients play important roles in the growth and development of most plant species. However, in perennial trees, the function of nutrients in different genotypes is poorly understood. Three different nutrient levels (low, sufficient, and high nutrient levels) were applied to two contrasting *Eucalyptus urophylla* cultivars (a high-growth cultivar ZQUA44 and a low-growth cultivar ZQUB15), and growth and expression levels were analyzed. Although the growth traits of both genotypes under nutrient starvation treatment were much lower than under abundant nutrients, tree height, crown width, and biomass of different ZQUA44 tissues were much higher than those of ZQUB15 at all three nutrient levels. Differentially expressed genes (DEGs) clustered into six subclusters based on their expression patterns, and functional annotation showed that the DEGs involved in glutathione metabolism and flavonoid biosynthesis may be responsible for nutrient starvation across different genotypes, while the DEGs involved in carotenoid biosynthesis and starch and sucrose metabolism may have a range of functions in different genotypes. The DEGs encoding the MYB-related family may be responsible for nutrient deficiency in all genotypes, while B3 may have different functions in different genotypes. Our results demonstrate that different genotypes may form different pathways to coordinate plant survival when they face abiotic stresses.

## 1. Introduction

Water, mineral nutrients, and light are the main external inputs needed by plants to grow. In southern China, which has an abundance of rainfall and sunlight, mineral nutrients are the essential limiting factor for plant growth and production. For example, the macroelement nitrogen (N) is one of the most widely distributed nutrients in plants and is an essential nutrient for plant growth. N is the most fundamental elemental constituent of proteins [1], and its deficiency causes a decrease in amino acids, resulting in reduced plant growth. Long-term N deficiency leads to a decreased leaf area, resulting in yellowing of the leaves and a lower photosynthesis rate, reducing crop yield [2]. Furthermore, phosphorus (P), potassium (K), calcium (Ca), magnesium (Mg), and sulfur (S) are fundamental elements in plant cells and are incorporated into proteins, genetic materials, and membranes [1]. The macroelements P, Mg, and Ca also play important roles in energy metabolism, enzyme activity regulation, and phytohormone signal transduction [1]. Therefore, the absorption of sufficient mineral nutrients is essential for plant growth. However, in many areas of the world, agricultural, horticultural, forest, and herb plantations are deficient in elements that support healthy and productive plant growth. Therefore, fertilizers are applied to maximize yields. However, different genotypes have different growth traits due to variations in mineral nutrient use efficiency (NUE), even when the same level of nutrient is applied. Trees with a high growth performance tend to be more efficient at absorbing and utilizing nutrients than low-growth individuals under the same cultivation conditions; this difference may be due to variations in genetic signature [3,4]. Consequently, the development of tree species with a higher NUE will greatly increase the input-output (i.e., fertilizer application–wood production) of plantations and prevent nutrients from being released into ecosystems. Thus, to develop effective breeding programs, it is vital to fully understand the underlying genetic basis of the high- and low-growth traits of trees in response to nutrient supply.

In recent years, there has been substantial progress in next-generation sequencing accompanying bioinformatics analysis, which has allowed the identification of key genes and critical pathways underlying the responses of various plant species to variable levels of nutrients [5]. In *Arabidopsis*, the response to nutrient deficiency involves various complex networks, including the rearrangement of gene expression, hormone signal transduction, and physiological and morphological modifications [6]. Tao et al. (2013) studied the effects of nutrient starvation on starch accumulation in *Landoltia punctata* and found that it inhibited universal metabolism. Furthermore, the expression of genes encoding the key enzymes involved in starch biosynthesis was increased, while that of those that play an important role in starch consumption was inhibited [7]. However, few nutrient-starvation studies have been conducted on perennial plant species, which have much larger genomes than herbaceous plants. In one such study, the genetic mechanism controlling the “stay-green” phenomenon in *Litchi chinesis* pericarp under foliar Mg treatment was investigated via de novo transcriptome sequencing [8]. The authors reported that DEGs were enriched in flavonoid biosynthesis, anthocyanin biosynthesis, and the abscisic acid (ABA) signal pathway, indicating the role of Mg in many metabolic pathways in litchi. Still, the molecular basis underlying differences between high- and low-growth genotypes of lignocellulose tree species in response to nutrient stress is unclear. It is necessary to identify nutrient-responsive genes and to clarify the regulatory and metabolic mechanisms that allow tree species to adapt during periods of nutrient deficiency.

*Eucalyptus* is one of the most important commercial tree species in the world and is widely grown for pulp production [9]. By 2017, about 4.6 million hectares of *Eucalyptus* had been planted in southern China, accounting for 6.5% of all forestry plantations and producing 30 million m^3^ of wood products (26.9% of the country’s annual wood production). *E. urophylla* is one of the most widely planted *E. urophylla* species in southern China due to its high growth rate. A high growth rate requires high nutrient availability, and therefore, the availability of essential nutrients is a crucial factor determining the wood production of tree species worldwide. The seedling stage is a critical period for the survival and establishment of trees [10]. Previously, we conducted an 18-month precision fertilizer control experiment on 100 *E. urophylla* clones and found that the biomass of some clones was significantly higher than that of other clones under the same nutrient level. Which genes lead to the differences between clones? What are the functions of these genes? These are the questions we aim to unravel.

Therefore, in this study, we analyzed the phenotypic responses of *E. urophylla* seedlings subjected to nutrient stress. Transcriptomic analyses were performed to study gene expression patterns under different nutrient stress conditions using RNA extracted from the leaves of 18-month-old seedlings. The main objective was to identify genes that were differentially expressed under control and stress conditions and to provide information regarding the molecular mechanism involved in nutrition treatment that can be used in future studies.

## 2. Materials and Methods

### 2.1. Plant Materials and Nutrient Treatments

The plants used in this study were grown from seeds obtained commercially from the Commonwealth Scientific and Industrial Research Organisation (CSIRO), Australia. All plants were cloned by tissue culture. Based on field studies, two *E. urophylla* genotypes were identified for use in the study: a high-growth cultivar (ZQUA44) and a low-growth cultivar (ZQUB15) (Yang et al., unpublished). Healthy seedlings of similar size were selected for each genotype and cultured in cylindrical containers with black-and-white film for moisture control. These were exposed to three nutrient treatments (low, sufficient, and high nutrient levels) under natural conditions in March. The low treatment entailed only one application of base fertilizer (250 g calcium-magnesium phosphate fertilizer (Norsterra, Norway)) per tree. The sufficient treatment included the application of base fertilizer and an after fertilizer (250 and 100 g compound fertilizer in August and the next March, respectively) per tree. The high treatment involved the base fertilizer (250 g calcium-magnesium phosphate fertilizer and 100 g compound fertilizer) and an after fertilizer (100 g urea in May and 150 and 100 g compound fertilizer in August and the next March, respectively) per one tree. Each treatment was replicated three times. After 18 months of culture, the leaves of all treatments were sampled and stored in liquid nitrogen.

### 2.2. Determination of Growth Characteristics

In total, three features were quantified in 18-month-old *E. urophylla* seedlings, with three biological replicates used for each treatment. Height was measured as the distance from the rhizome on the ground to the shoot apex and was measured using a height gauge. The ground diameter was determined at the base of a tree shoot using calipers. Crown width was the width of the north-south or east-west direction of trees and was determined by a scaled measuring stick. The biomass of the different treatments referred to the dry weight of different tissues, including leaves, stems, and branches. All tissues were sampled and dried in a drying oven. Each sample was measured until a constant weight was achieved.

### 2.3. RNA Isolation, Sequencing, and Assembly

Total RNA was isolated from the leaves of the three replicate plants in each treatment using the Qiagen RNAeasy kit (Qiagen China, Shanghai, China) and purified using an RNAclean Kit (Tiangen Biotech (Beijing) CO. LTD., China) following the manufacturer’s instructions. The integrity of the RNA was monitored on 1% agarose gels, and the purity was checked using a NanoPhotometer^®^ spectrophotometer (Implen, Westlake Village, CA, USA). The RNA concentration was determined using a Qubit^®^ RNA assay kit with the Qubit^®^ 2.0 fluorometer (Life Technologies, San Francisco, CA, USA). A total amount of 3 µg high-quality RNA per sample was used for subsequent RNA sequencing. The cDNA library was constructed for each of the nine RNA samples and sequenced on the Illumina HiSeq 2500 platform (Illumina Inc., San Diego, CA, USA). Before assembly, the adapter sequences, poly N, and low-quality reads were removed from the raw data. An index of the reference genome (directly downloaded from https://phytozome.jgi.doe.gov/pz/portal.html#!bulk?org=org_egrandis, accessed on 19 August 2020) was built using Bowtie v2.0.6 and the paired-end clean reads were aligned to the reference genome using TopHat v2.0.9 [11]. Then, the mapped reads of each sample were assembled using both Scripture (beta2) [12] and Cufflinks (v2.1.1) [13].

### 2.4. Normalization of Gene Expression Levels and Identification of DEGs

To evaluate gene expression levels, the paired-end clean reads that were mapped to the reference genome were used for the FPKM calculation of each sample using Cuffdiff [13]. The FPKM was calculated based on the length of the fragments and the reads count mapped to the fragment.

To distinguish the transcriptional changes under the different treatments in the two genotypes, the DEGs under the different treatments were identified by comparing the expression levels under the sufficient and low treatments to those under the high and sufficient treatments in ZQUA44 and ZQUB15, respectively, using the DESeq2 R package (1.16.1). To eliminate false positives, the false discovery rate (FDR) was calculated to adjust the threshold of the *p*-value. Transcripts with a minimal two-fold difference in expression (|log_2_Fold change| ≥ 1) and an FDR ≤ 0.01 were considered to be differentially expressed between the three treatments. For convenience, DEGs with higher expression levels under the sufficient treatment than under the low treatment, as well as those with higher expression levels under the high treatment than under the sufficient treatment, were considered upregulated, whereas those in opposition were considered downregulated.

To assess the gene expression patterns under different nutrient conditions within each genotype, expression pattern analysis was performed, which assigned all DEGs of ZQUA44 and ZQUB15 across the two treatment levels to nine expression profiles, using Short Timeseries Expression Miner (STEM) version 1.3.8 (Ernst and Bar-Josepheight, 2006). The DEGs belonging to the same cluster were proposed to have a similar expression pattern. For each genotype, the clustered profiles of DEGs with *p* < 0.05 were considered to be significantly different from the reference set.

#### 2.4.1. Functional Annotation and GO and KEGG Classification

The identified genes were annotated by referring to E. grandis in the Phytozome plant genomics resource (https://phytozome.jgi.doe.gov/pz/portal.html#!bulk?org=org_egrandis, accessed on 19 August 2020) and The Arabidopsis Information Resource (TAIR) (https://www.arabidopsis.org/, accessed on 19 August 2020). Then, GO terms were determined by AgriGO (http://bioinfo.cau.edu.cn/agriGO/index.php, accessed on 25 August 2020) with Arabidopsis as the background and an FDR < 0.05 was set as the threshold. The KEGG Orthology-Based Annotation System (KOBAS) 3.0 tool (http://kobas.cbi.pku.edu.cn/index.php, accessed on 26 August 2020) was used to analyze the potential functions of the target genes in the pathways under the three different nutrition treatments (*p* < 0.01).

#### 2.4.2. Validation of the Expression Level

Ten genes with different expression patterns revealed by RNA sequencing were randomly selected for validation by reverse transcription quantitative real-time PCR (qRT-PCR). RNA extracted from leaves from all samples was used for qRT-PCR validation. cDNA was synthesized using a Tiangen FastKing RTKit (Tiangen Biotech). Gene-specific primers for qRT-PCR were designed based on the corresponding sequence on the NCBI Primer-BLAST (https://www.ncbi.nlm.nih.gov/tools/primer-blast/index.cgi?LINK_LOC, accessed on 27 August 2020) and are listed in Table S1. Actin (EF145577) was used as an internal control. The qRT-PCR was performed using the FastKing RT Kit and determined using an Applied Biosystems 7500 fast real-rime PCR system following the manufacturer’s instructions. Three technical replicates were performed for each gene. A regression analysis was performed between qRT-PCR and RNA sequencing, including all of the genes of the two genotypes at the three different treatments using the r package (version 3.1.3, http://cran.r-project.org/, accessed on 15 September 2020).

## 3. Results

### 3.1. Effect of Different Nutrient Treatments on Tree Growth Characteristics

**Figure 1:**
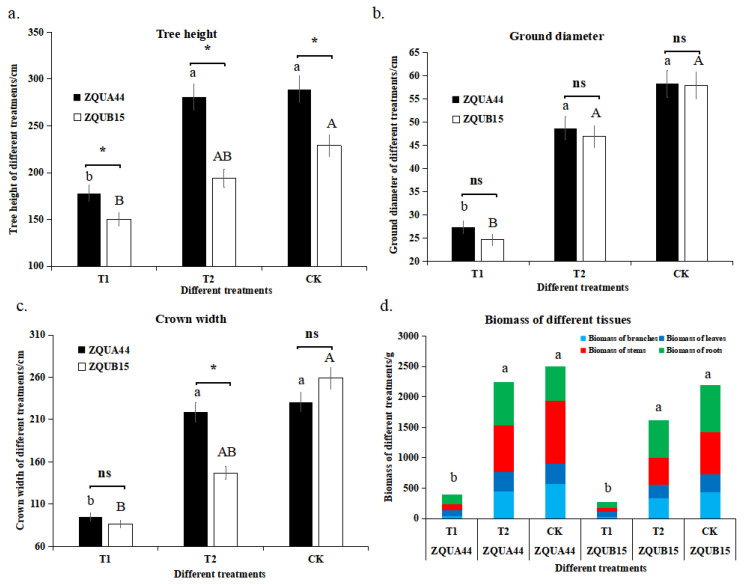
The growth traits at low nutrient application levels (T1), sufficient nutrient level (T2), and high nutrient level (CK) in genotypes ZQUA44 and ZQUB15. (**a**) tree height; (**b**) ground diameter; (**c**) crown width; (**d**) biomass of different treatments in different genotypes. The lowercase letter ab and the capital letter AB represent the significant difference between the treatments of ZQUA44 and ZQUB15, respectively. The “*” indicates the significant difference between ZQUA44 and ZQUB15 at the same treatments. The “ns” indicates the no significant difference were found between ZQUA44 and ZQUB15.

To examine the effects of different long-term nutrient treatments on the high- and low-growth genotypes of *E*. *urophylla*, we measured growth traits, including tree height, ground diameter, and crown width, as well as the biomass of different tissues (e.g., the biomass of branches, leaves, roots, and stems) 18 months after nutrient treatment. There were significant differences (*p* < 0.05 unless otherwise stated) in all growth traits between the different treatments (Figure 1). For example, tree heights of the high- and low-growth genotypes under ‘sufficient’ (see Methods) nutrient levels (281.00 and 194.67 cm, respectively) and ‘high’ nutrient levels (289.00 and 229.00 cm, respectively) were significantly greater than under low nutrient levels (178.00 and 150.00 cm, respectively) (Figure 1). Moreover, the leaves under low nutrient levels were much smaller (and showed signs of disease) compared to those under sufficient and high treatments (Figure S1). No significant differences in height were found between the high and sufficient treatments for ZQUA44, while a significant difference was observed for ZQUB15. The same was found in regard to increases in ground diameter and crown width. To further evaluate the effects of nutrients on plant growth, the biomass of different tissues was assessed. Stems, branches, roots, and leaves (and overall biomass) were much lower under low nutrient levels than in the other treatments for both genotypes, indicating that nutrient availability was the most important factor that restricted plant growth.

However, the growth traits of the different genotypes varied at the same level of nutrient treatment. For example, ZQUA44 plants were much taller than ZQUB15 plants at all three nutrient levels. The same was observed for ground diameter. Interestingly, the crown width was similar under the low and sufficient treatments, while a much larger crown was observed under high nutrients. All biomass values were greater for ZQUA44 than for ZQUB15 under low nutrient levels, indicating that the genetic basis of the different genotypes was another important factor causing variation in phenotypic performance. The differences in plant growth traits between the high and low nutrient applications were much larger in ZQUA44 than in ZQUB15. In addition, no significant differences were observed in height between the sufficient and high treatments in ZQUA44, indicating a higher NUE in ZQUA44.

### 3.2. An RNA Sequencing Approach for the Assembly, Quantification, Identification, and Clustering of DEGs in Response to Nutrient Treatments

**Figure 2:**
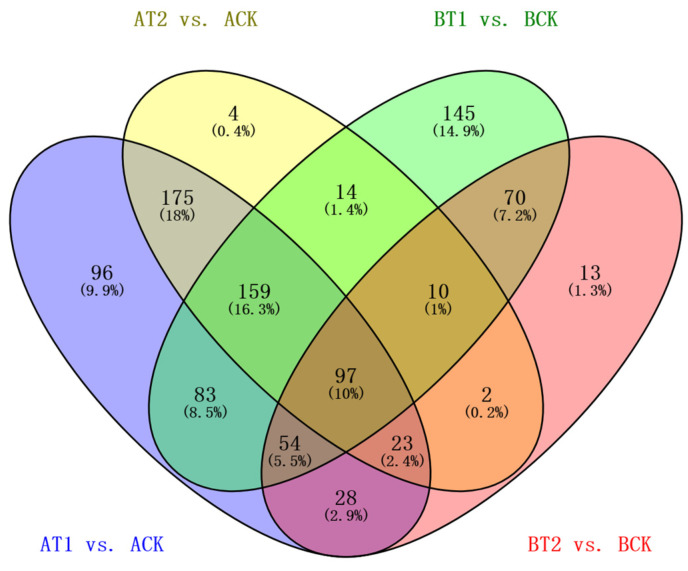
Venn analysis of different genotypes at different treatments. A: ZQUA44; B: ZQUB15. T1: treatment of low nutrient level; T2: treatment of sufficient nutrient level; CK: treatment of high nutrient level. AT1 indicates the treatment of low nutrient levels in genotype ZQUA44; AT2 indicates the treatment of sufficient nutrient levels in genotype ZQUA44; ACK indicates the treatment of high nutrient levels in genotype ZQUA44; BT1 indicates the treatment of low nutrient levels in genotype ZQUB15; BT2 indicates the treatment of sufficient nutrient level on genotype ZQUB15; BCK indicates the treatment of high nutrient level on genotype ZQUB15. The labels are the same as below.

To evaluate the genetic variation in the two genotypes under different treatments, transcriptome profiling of 18 samples of the two genotypes was conducted on an Illumina Hiseq 2500 platform. In total, 8.42–11.11 million 125 bp pair-end reads were generated (Table 1). After removing adapter, ploy-N, and low-quality reads using in-house perl scripts, 8.15–10.72 million clean reads were obtained. Then, clean data with a high Q20 (94.43–98.41%) and Q30 (85.65–95.71) and low error rate (<0.03%) were used for alignment to the reference genome using TopHat v2.0.9. A range of 3.21–5.72 million reads were mapped to the reference genome, and more than 96% of them were uniquely mapped. The expression levels of protein-coding genes were represented by fragments per kilo-base of exon per million fragments mapped (FPKMs). The genes with FPKMs < 0.1 in all samples were filtered out, and the remaining genes were used for further analysis. To detect genes that were specifically expressed under different treatments, the DESeq2 R package (1.16.1) was used to analyze samples of the two genotypes under the different treatments. In total, 973 DEGs were detected in ZQUA44 and ZQUB15 under the low and sufficient nutrient treatments compared to high treatment using a threshold of a 2-fold change in gene expression, as previously applied when using the degR package (*p* < 0.01, and *Q* < 0.10, Figure 2). Of these DEGs, 470 were found in both ZQUA44 and ZQUB15, while 275 and 228 DEGs were found in only ZQUA44 and ZQUB15, respectively.

**Figure 3:**
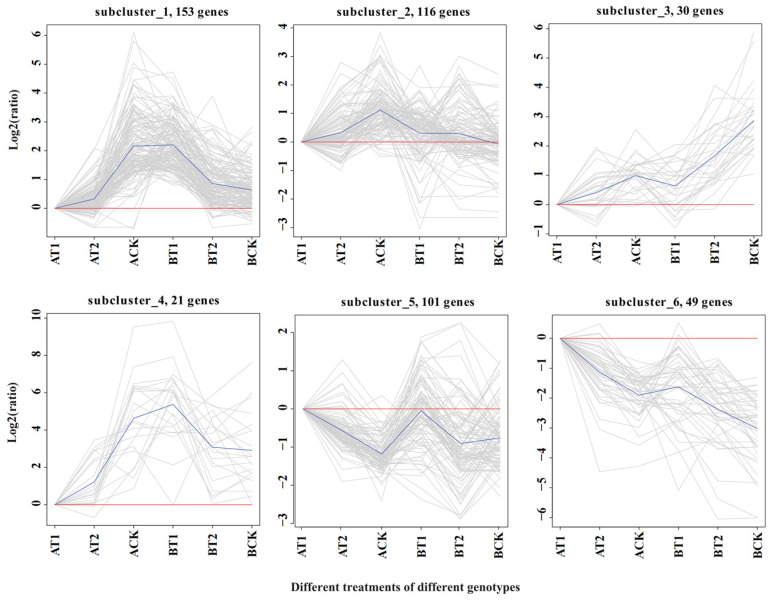
Cluster analysis of the common differentially expressed genes in ZQUA44 and ZQUB15. The ordinate is the value of the expression value after logarithmic centralization correction and the red lines indicate the value is zero. The blue lines represent the relative corrected gene expression levels of all genes in this cluster under different experimental conditions.

To further determine the possible mechanisms through which the different genotypes respond to different nutrient levels, the expression trends of common DEGs between the two genotypes were analyzed using ggplot2 package implemented in R (Version 3.0.3). The DEGs clustered into six subclusters, which were divided into three groups: Group 1 (G1) consisting of subclusters 1 (153 DEGs), 2 (116 DEGs), and 4 (21 DEGs); Group 2 (G2) consisting of subcluster 3 (30 DEGs); and Group 3 (G3) consisting of subclusters 5 (101 DEGs) and 6 (49 DEGs) (Figure 3). The DEGs in G1 were all activated under nutrient deficiency in genotype ZQUB15, while they were inhibited in genotype ZQUA44. The DEGs in G2 were all inhibited under nutrient deficiency, while they were activated under high nutrient levels in both genotypes. The opposite situation was observed in G3, in which the DEGs were all activated under nutrient deficiency, while they were inhibited under high nutrient levels in both genotypes. Because the same DEGs in the different genotypes had different expression patterns in G1, we propose that the DEGs in this group may be responsible for the genotype/nutrient interaction. In Groups 2 and 3, the DEGs were activated or inhibited in both genotypes. We therefore propose that the DEGs of these groups may be responsible for the nutrient responses across clones. The DEGs found only in either ZQUA44 or ZQUB15 may be genotype-specific genes between low- and high-performing clones.

### 3.3. Functional Enrichment of the DEGs of Different Genotypes in Response to Nutrient Treatments

**Figure 4:**
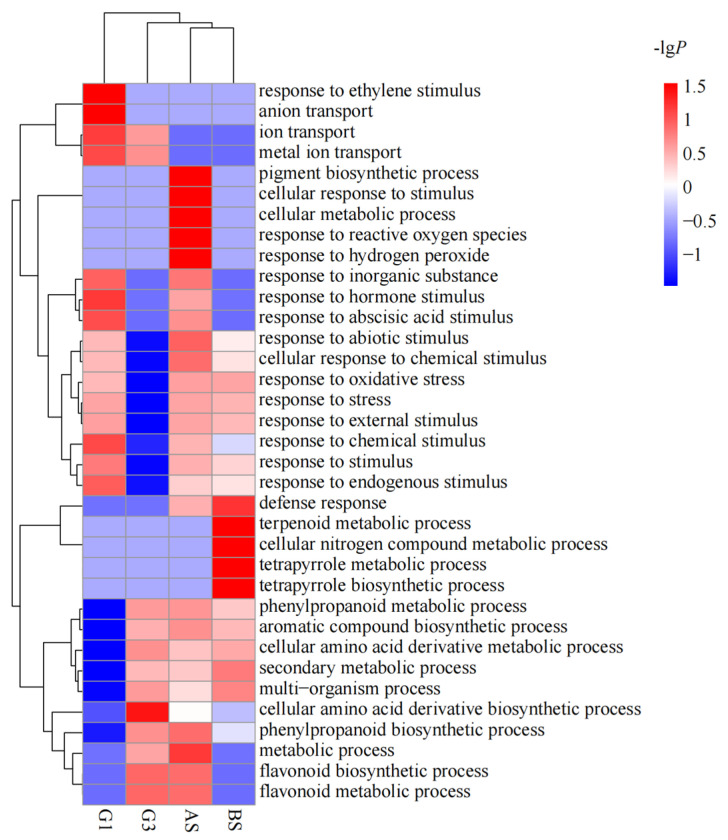
Gene Ontology analysis of differentially expressed genes (DEGs) from different expression groups. G1 indicates the DEGs inhibited at low nutrient treatment in ZQUA44 and activated in ZQUB15; G3 implicates the DEGs activated at low nutrient treatment in both genotypes; AS represents DEGs identified in ZQUA44; BS indicates DEGs identified in ZQUB15. The *p*-values were used for the heatmap after the calculation by −lg*P*. Red indicates the higher significance of the GO term, and blue indicates the lower significance of the GO term.

Gene ontology (GO) classifications were determined to analyze the possible functions of DEGs that were differentially expressed in the ZQUA44 and ZQUB15 genotypes and the common DEGs that were found in both genotypes (Figure 4). A total of 35 GO terms in biological processes were identified in four groups of DEGs, including AS for DEGs that were differentially expressed in ZQUA44, BS for DEGs that were differentially expressed in ZQUB15, and G1 and G3 for the common DEGs (*p* < 0.0001, *Q* < 0.01). Overall, 12 of the significant GO terms identified in G1 were involved in responses to stress or stimulus. The most significant processes were the response to a stimulus and the response to a chemical stimulus. In G3, 57.1% of the 14 identified GO terms were involved in biosynthetic or metabolic processes, and only two GO terms were involved in responses to stress or stimulus. The most significant processes in this group were the flavonoid metabolic process, secondary metabolic process, and flavonoid biosynthetic process. In total, 27 GO terms were identified in the DEGs identified in ZQUA44, and 15 of them were involved in responses to stimulus or stress. The most significant terms were response to stimulus and response to abiotic stimulus in ZQUA44. In ZQUB15, 20 GO terms were identified, and 10 of them were involved in metabolic or synthesis processes. The most significant terms were response to stimulus and secondary metabolic process. Furthermore, the DEGs of all groups were involved in the cellular amino acid derivative biosynthetic process, phenylpropanoid biosynthetic process, response to chemical stimulus, and response to stimulus. Overall, 18 GO terms for molecular functions were found in the five groups of DEGs (Table 2). DEGs with oxidoreductase activity and catalytic activity were significantly enriched in all five groups. Those involved in transcription activator activity were significantly enriched in G1, those involved in transporter activity were significantly enriched in AS, and those involved in transferase activity were significantly enriched in BS.

A Kyoto Encyclopedia of Genes and Genomes (KEGG) analysis showed that the DEGs involved in metabolic pathways were significantly enriched in G1, G3, and AS (Table 3, *Q* < 0.2). The DEGs in G1 were involved in amino acid metabolism, including ascorbate and aldarate metabolism, arginine and proline metabolism, tyrosine metabolism, galactose metabolism, and alanine, aspartate, and glutamate metabolism. Those involved in plant hormone signal transduction, galactose metabolism, protein processing in endoplasmic reticulum, nitrogen metabolism, cysteine and methionine metabolism, and glycerolipid metabolism were significantly enriched in AS. Those in BS were involved in biosyntheses, such as cutin, suberine, wax biosynthesis, and zeatin biosynthesis. The most significant processes were phenylpropanoid biosynthesis and sulfur metabolism in G2, while glutathione metabolism; phenylalanine, tyrosine, and tryptophan biosynthesis; and stilbenoid, diarylheptanoid, and gingerol biosynthesis were most significantly enriched in G3.

### 3.4. DEGs Involved in Plant Hormone Signal Transduction

**Figure 5:**
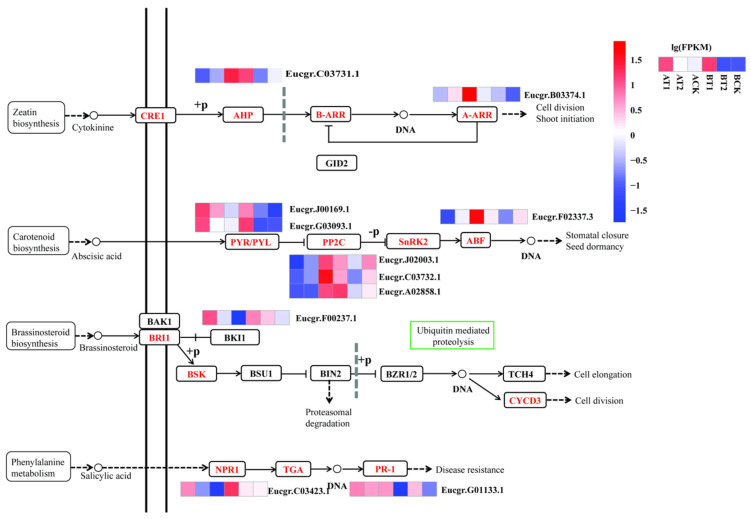
Analysis of important plant hormone signal transduction pathways related to nutrient treatment. The normalized expression levels were used for heatmap visualization. Red indicates the higher expression levels of DEGs, and blue indicates the lower expression levels of DEGs.

In total, 11 DEGs were identified as being involved in plant hormone signal transduction, including the signal transduction pathway of cytokinine, abscisic acid (ABA), brassinosteroid, and salicylic acid (Table 4, Figure 5). Half of them were enriched in the ABA signal transduction pathway, including two DEGs encoding PYR/PYL (ABA receptor), three encoding P2C (protein phosphatase 2C), and one encoding ABF (ABA-responsive element binding factor). The DEGs involved in plant hormone signal transduction had different expression patterns. For example, those encoding NPR1 (nonexpresser of pathogenesis-related genes), BKI1 (A leucine-rich repeat receptor serine/threonine kinase that could perceive brassinosteroids at the plasma membrane called BRI1, and the BRI1-interacting protein called BKI1), and PYR/PYL were all activated under nutrient deficiency in both genotypes, while those encoding AHP (histidine-containing phosphotransfer peotein, *Eucgr.J00169.1*, and *Eucgr.G03093.1*) and PP2C (*Eucgr.J02003.1*, *Eucgr.C03732.1*, and *Eucgr.A02858.1*) were activated under high nutrient levels in the high-growth ZQUA44 genotype but were inhibited under low nutrient levels in the low-growth ZQUB15 genotype. The DEGs encoding A-ARR (Eucgr.B03374.1) and ABF (*Eucgr.F02337.3*) were activated under high nutrient levels in ZQUA44, while there were no significant differences observed in ZQUB15.

### 3.5. Transcription Factors (TFs) Responding to Nutrient Deficiency

To identify the important TFs responsible for nutrient deficiency, the DEGs from different groups were annotated in the plant transcription factor database (Table 5). In total, 74 DEGs encoding 21 TF families were obtained in all groups in ZQUA44 and ZQUB15. Of all groups, G1 had the most abundant DEGs encoding 10 TFs, followed by AS, which had 25 DEGs encoding 15 TFs. The most abundant TFs were ERF (10) and MYB (10), followed by NAC (8) and WRKY (7). Seven DEGs encoding ERF and six encoding NAC were found in G1, while five encoding WRKY were found in BS. Some TF families were only found in one group; for example, the TF of MIKC_MADS was only found in G3, the TFs of CPP and RAV were only found in BS, the TFs of LBD and SBP were only found in G1, and the TFs of NF-YA, TCP, bZIP, Dof, and B3 were only found in AS.

Further analysis of WRKY encoding genes showed that two DEGs encoding WRKY23 (*Eucgr.H00996*) and WRKY75 (*Eucgr.B03520*) were inhibited at the low nutrient level, while the other five DEGs encoding WRKY26 (*Eucgr.B04010*), WRKY33 (*Eucgr.K02940*), WRKY50 (*Eucgr.C00675*), WRKY6 (*Eucgr.E04011*), and WRKY75 (*Eucgr.I01633*) were all activated at the low nutrient level (Table 6).

### 3.6. Validation of RNA Sequencing Results via Quantitative Real-Time Polymerase Chain Reaction (qRT-PCR)

**Figure 6:**
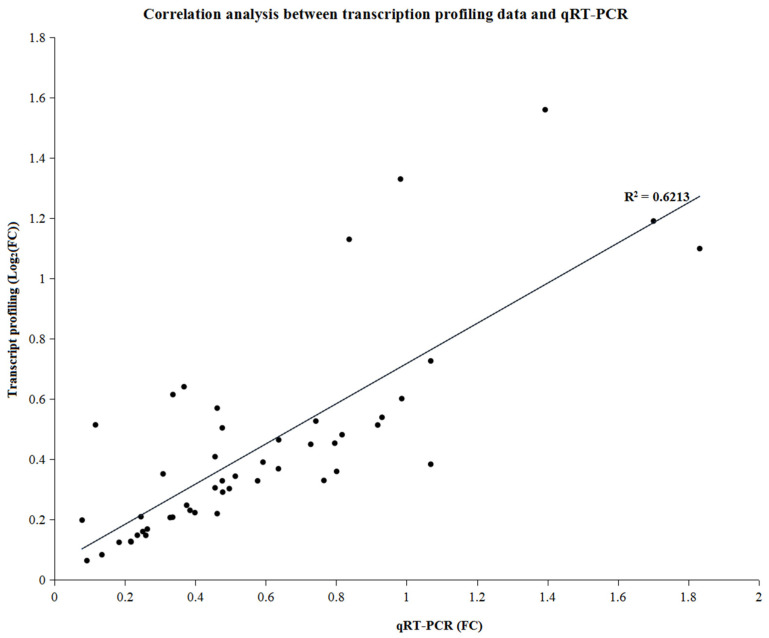
Correlation analysis between RNA sequencing results and quantitative real-time polymerase chain reaction.

To validate the RNA sequencing results, 10 genes with different expression patterns were randomly selected for qRT-PCR using an Applied Biosystems 7500 fast real-rime PCR system (Applied Biosystems, Waltham, MA, USA). A similar expression pattern was found for all three nutrient treatments for both genotypes when the qRT-PCR results and RNA sequencing data were compared, indicating that the expression results generated by RNA sequencing were reliable and could be used for further study (R^2^ = 0.6213, Figure 6).

## 4. Discussion

Comparative transcriptome analyses are powerful for analyzing the genotypes of plants that exhibit different phenotypes under various external treatments [14]. In this study, we compared the transcriptomic differences between two *E. urophylla* cultivars that show different phenotypes under different nutrient application levels. The phenotypic characteristics exhibited a similar tendency in the two *E. urophylla* genotypes at low, sufficient, and high nutrient levels. For example, tree height, ground diameter, crown width, and the biomass of different tissues were much lower in both genotypes under low nutrient conditions than under sufficient and high levels, indicating that nutrient availability was the most important factor restricting plant growth, which agreed with previous studies that nutrient limitation significantly restricted plant growth and physiological metabolisms [15,16].

However, the growth traits were much higher for ZQUA44 than for ZQUB15 in all three nutrient levels, indicating that trees with different genotypes may respond differently. This result agrees with prior research on *Oryza sativa*, *Vigna radiata*, and *Zea may* that different individuals have different production under the same nutrient levels [17,18,19,20]. To address this further, we conducted comparative transcriptomic sequencing analyses. The identified DEGs were clustered into different groups with different functions. The DEGs had the same expression pattern in both genotypes and were significantly enriched in glutathione metabolism, flavonoid biosynthesis, and metabolic or biosynthetic processes, indicating that these DEGs may have similar functions when there are underlying nutrient deficiency stresses across clones [21]. The other DEGs had contrasting expression patterns in the two genotypes and were significantly enriched in response to stimulus or stress, indicating that they may be responsible for the nutrient/genotype interaction. Furthermore, the DEGs that were differentially expressed in AS were significantly enriched in response to stimulus or stress, while those in BS were significantly enriched in metabolic or biosynthetic processes, which implies that different genotypes had different DEGs to respond to the same abiotic stresses. DEGs associated with oxidoreductase activity and catalytic activity were also abundant in all five groups. This indicates that different genotypes had similar biological responses when exposed to abiotic stress, although different DEGs were involved. Our results are consistent with the previous study that drought stress induces different key genes and pathways in two wheat (*Triticum aestivum* L.) varieties [21].

Phytohormones are the key regulators of plant growth and development and are mediators of environmental stress responses [6]. Abscisic acid (ABA) is an essential phytohormone that controls many developmental stages, including seed dormancy, seedling development, flowering, and fruit ripening. ABA levels increase to adapt to abiotic stresses, such as drought, high temperature, chilling, and salinity. Plants can translate environmental stress into a physiological response through ABA signal transduction. Five groups of proteins have been identified in this pathway, including the ABA receptor PYL/PYR protein, PP2C protein, and kinase family SnRK2. P2C, which is a negative regulator of ABA signaling pathways, positively regulates the abiotic stress signaling pathway in herbaceous plants [22]. In our study, three DEGs encoding the PP2C protein were inhibited under nutrient deficiency in ZQUA44, whereas they were activated in ZQUB15. Furthermore, two DEGs encoding the negative regulator of PYL/PYR protein were identified, and both were activated at low nutrient levels in both genotypes. Because the PYL/PYR protein is a negative regulator of PP2C protein, the expression level of *PP2C* agreed with the pattern in ZQUA44, but this did not occur in ZQUB15, indicating that the genetic variation in the two genotypes may be the reason for the phenotypic variation [21,23].

In addition to phytohormones, many TFs control signal transduction under biotic and abiotic stresses, including the MYB (10), ERF (10), NAC (8), WRKY (7), MYB-related (6), and bHLH (5) families, whose members promote or suppress abiotic stress responses [24,25,26,27,28]. For example, MYBs have been studied in various plant species and have been proven to be related to growth and development and stress responses in plants [29,30,31,32,33,34]; these results agreed with our study that MYB-encoding DEGs may be involved in resistance to nutrient deficiencies. Ten DEGs encoding ERF were obtained in our study, and seven of them were enriched in G1, indicating their role in the nutrient/genotype interaction. Our results are consistent with previous studies that ERFs are one of the most important TF families, and they are involved in various plant-specific processes, such as developmental processes, regulation of metabolism, and the response to biotic and abiotic stresses [35,36]. Members of the WRKY family are essential regulators of plant innate immunity, and they play key roles in regulating biotic and abiotic stress reactions in various plant species [7,37]. Furthermore, a gene encoding WRKY in *Larrea tridentata* was proven to activate ABA signaling, which plays an essential role in stress resistance [38,39,40,41,42,43,44,45,46]. In poplar, 15 *PtWRKYs* in leaves were identified as involved in the nitrogen response, and *PtWRKY33* was down-regulated in the nitrogen deficiency group [47]. In our study, a DEG encoding *WRKY33* was activated in the nutrient deficiency treatment, which was inconsistent with the result in poplar, indicating that homologous genes in different plant species may have different functions. A gene encoding TCP, which plays a key role in cell proliferation in developing tissues and is predicted to be related to tree height growth, was inhibited at low nutrient levels in ZQUA44, which may explain why the tree height of ZQUA44 was restricted to a greater extent than that of ZQUB15 under low nutrient levels [48].

In addition to the phytohormone-related genes and TFs, other genes encoding F-box protein, late embryogenesis abundant (LEA) protein, ammonium transporter 2, cytochrome P450, UDP-glycosyltransferase (UGT), and phenylalanine ammonia-lyase were also identified in this study. F-box protein is related to cell cycle regulation and signal transduction [49]. Several late embryogenesis proteins have been found to be related to resistance to water deficiency; they function by protecting cytoplasmic components [50]. The identification of this gene in our study provides new insight into its possible functions in resistance to nutrient deficiency. Genes encoding ammonium transporter 2 were significantly activated under high nutrient levels in ZQUA44, while no significant differences were observed in ZQUB15, indicating that a higher nutrient level may promote the transport ability of mineral elements in ZQUA44 to maintain their high growth ability [51,52,53]. UGTs can catalytically transfer the glycosyl group from activated donor molecules to specific receptor molecules, and they play important roles in various processes, such as hormone signal transduction and cell wall polysaccharide synthesis [54]. UGTs, together with phytohormones, play a pivotal role in stress resistance by changing the water solubility of the receptor molecule [55,56]. In our study, three DEGs were activated in ZQUA44 and ZQUB15; two were activated under nutrient deficiency in ZQUA44, while six were activated in ZQUB15, indicating that different members of this gene family may play different roles in different genotypes.

## 5. Conclusions

Our results clearly demonstrate that not only the environmental factors but also the genetic differences play essential roles in tree performance. High-growth genotype ZQUA44 and low-growth genotype ZQUB15 exhibited significant differences in their responses to nutrient starvation in terms of tree height, crown width, and the biomass of different tissues. Transcriptomic profiling indicated that the DEGs showed similar expression trends that may be responsible for resistance to nutrient starvation across genotypes. The different genotypes had different genes that responded to the treatment, and the same genes had different expression patterns between the genotypes. The DEGs showed opposite expression trends, which may be responsible for the nutrient/genotype interaction. The DEGs in AS and BS may be genotype-specific; they were involved in response to stimulus or stress in ZQUA44 and were involved in metabolic or synthesis processes in ZQUB15. These findings provide a genetic basis for the breeding of *E. urophylla* with tolerance to nutrient deficiency.

## Figures and Tables

**Table 1:** Sequencing and assembly statistics for the nine transcriptome data of two *E. urophylla* genotypes of nutrient treatment.

Sample	ID	No. of Raw Reads (10^7^)	No. of Clean Reads (10^7^)	No. of Clean Basepairs (10^6^)	No. of Mapped Reads (10^7^)	Uniquely Mapped (10^7^)	Mapped Percentage (%)
ZQUA44	T1_1	9.49	9.18	13.55	5.13	5.00	54.48
ZQUA44	T1_2	9.06	8.78	13.11	4.77	4.63	52.76
ZQUA44	T1_3	9.73	9.49	14.24	4.09	3.94	41.53
ZQUA44	T2_1	9.60	9.28	13.67	4.91	4.79	51.58
ZQUA44	T2_2	9.35	9.08	13.57	3.79	3.62	39.82
ZQUA44	T2_3	10.47	10.11	15.17	5.46	5.32	52.66
ZQUA44	CK_1	10.28	9.89	14.85	5.72	5.57	56.27
ZQUA44	CK_2	9.18	8.84	13.07	4.51	4.33	48.91
ZQUA44	CK_3	8.81	8.58	12.82	3.63	3.50	40.75
ZQUB15	T1_1	9.83	9.51	14.05	4.09	3.93	41.35
ZQUB15	T1_2	9.70	9.34	13.95	4.70	4.56	48.81
ZQUB15	T1_3	8.42	8.15	12.21	3.37	3.26	40.01
ZQUB15	T2_1	9.58	9.22	13.59	4.99	4.87	52.75
ZQUB15	T2_2	8.76	8.52	12.73	3.36	3.24	37.98
ZQUB15	T2_3	10.58	10.30	15.37	4.30	4.15	40.34
ZQUB15	CK_1	8.47	8.22	12.27	3.21	3.07	37.37
ZQUB15	CK_2	8.97	8.68	12.96	4.29	4.19	48.24
ZQUB15	CK_3	11.11	10.72	16.04	5.11	4.99	46.64

**Table 2:** Annotation of molecular function of differentially expressed genes at different groups.

Groups of DEGs	GO Term	Description	Number of Enriched DEGs	*p*-Value	FDR
G1	GO:0016701	oxidoreductase activity, acting on single donors with incorporation of molecular oxygen	5	4.00 × 10^−6^	0.001
G1	GO:0003824	catalytic activity	80	0.0003	0.039
G1	GO:0016757	transferase activity, transferring glycosyl groups	11	0.00045	0.039
G1	GO:0016563	transcription activator activity	6	0.00074	0.048
G2	GO:0016491	oxidoreductase activity	7	1.90 × 10^−5^	0.0001
G2	GO:0003824	catalytic activity	13	0.0015	0.0042
G3	GO:0003824	catalytic activity	66	7.00 × 10^−12^	1.20 × 10^−9^
G3	GO:0016787	hydrolase activity	27	1.10 × 10^−5^	0.00094
G3	GO:0016491	oxidoreductase activity	16	1.80 × 10^−5^	0.0011
G3	GO:0004091	carboxylesterase activity	8	3.40 × 10^−5^	0.0015
AT	GO:0003824	catalytic activity	96	8.50 × 10^−8^	2.40 × 10^−5^
AT	GO:0016491	oxidoreductase activity	25	4.90 × 10^−6^	0.00069
AT	GO:0005215	transporter activity	22	0.00014	0.013
AT	GO:0046527	glucosyltransferase activity	6	0.00024	0.017
AT	GO:0022892	substrate- transporter activity	17	0.00035	0.018
AT	GO:0016706	oxidoreductase activity, acting on paired donors, with incorporation or reduction of molecular oxygen, 2-oxoglutarate as one donor, and incorporation of one atom each of oxygen into both donors	5	0.00037	0.018
AT	GO:0022857	transmembrane transporter activity	17	0.0007	0.028
AT	GO:0016705	oxidoreductase activity, acting on paired donors, with incorporation or reduction of molecular oxygen	6	0.0016	0.047
AT	GO:0022891	substrate- transmembrane transporter activity	14	0.0017	0.047
AT	GO:0016758	transferase activity, transferring hexosyl groups	8	0.0014	0.047
BT	GO:0003824	catalytic activity	84	2.00 × 10^−8^	4.00 × 10^−6^
BT	GO:0016491	oxidoreductase activity	22	5.90 × 10^−6^	0.0006
BT	GO:0008194	UDP-glycosyltransferase activity	8	2.10 × 10^−5^	0.0014
BT	GO:0016758	transferase activity, transferring hexosyl groups	9	7.20 × 10^−5^	0.0037
BT	GO:0016757	transferase activity, transferring glycosyl groups	10	0.00052	0.021
BT	GO:0046527	glucosyltransferase activity	5	0.00074	0.025
BT	GO:0016209	antioxidant activity	5	0.0011	0.033
BT	GO:0016765	transferase activity, transferring alkyl or aryl (other than methyl) groups	5	0.0014	0.036

**Table 3:** Significant KEGG pathways enriched at different groups.

Groups	KEGG Pathway	ID	DEGs Number	*p*-Value	*Q*-Value
G1	Ascorbate and aldarate metabolism	ath00053	4	0.0002	0.0085
G1	Arginine and proline metabolism	ath00330	3	0.0024	0.0547
G1	Tyrosine metabolism	ath00350	2	0.0164	0.2424
G1	Galactose metabolism	ath00052	2	0.0260	0.2424
G1	Alanine, aspartate and glutamate metabolism	ath00250	2	0.0298	0.2424
G1	Limonene and pinene degradation	ath00903	1	0.0346	0.2424
G1	Monoterpenoid biosynthesis	ath00902	1	0.0377	0.2424
G1	Metabolic pathways	ath01100	17	0.0439	0.2471
G2	Phenylpropanoid biosynthesis	ath00940	2	0.0040	0.0202
G2	Sulfur metabolism	ath00920	1	0.0249	0.0623
G3	Phenylalanine, tyrosine and tryptophan biosynthesis	ath00400	2	0.0111	0.1867
G3	Stilbenoid, diarylheptanoid and gingerol biosynthesis	ath00945	1	0.0243	0.1867
G3	Metabolic pathways	ath01100	12	0.02515	0.1867
G3	Glutathione metabolism	ath00480	2	0.0325	0.1867
AS	Galactose metabolism	ath00052	3	0.0030	0.0938
AS	Plant hormone signal transduction	ath04075	6	0.0063	0.0938
AS	Protein processing in endoplasmic reticulum	ath04141	5	0.0072	0.0938
AS	Metabolic pathways	ath01100	21	0.0112	0.1088
AS	Nitrogen metabolism	ath00910	2	0.0288	0.1991
AS	Cysteine and methionine metabolism	ath00270	3	0.0306	0.1991
AS	Glycerolipid metabolism	ath00561	2	0.0414	0.2308
BS	Cutin, suberine and wax biosynthesis	ath00073	2	0.0076	0.1249
BS	Zeatin biosynthesis	ath00908	2	0.0109	0.1249

**Table 4:** Differentially expressed genes involved in plant hormone signal transduction pathways.

Gene ID	AT1 (FPKM)	AT2 (FPKM)	ACK (FPKM)	BT1 (FPKM)	BT2 (FPKM)	BCK (FPKM)	Uniprot	Symbol	Subcluster
*Eucgr.A01486*	0.64	0.87	6.35	4.08	2.92	4.94	Q5SN75	P2C08	sub4
*Eucgr.A02858*	0.24	0.30	9.65	10.76	1.75	3.41	Q9FLI3	P2C75	sub4
*Eucgr.C03732*	21.00	24.44	76.28	46.38	23.80	40.61	P49597	P2C56	sub1
*Eucgr.F00253*	0.55	0.51	0.00	0.70	0.00	0.00	Q9FX08	P2C12	sub6
*Eucgr.H04087*	16.26	226.00	35.9	19.74	15.81	14.61	Q3EAF9	P2C49	sub1
*Eucgr.J02003*	8.73	181.00	83.02	56.76	26.57	60.24	Q9ZW21	P2C24	sub4
*Eucgr.C03337*	1222.61	1033.70	638.04	37.29	57.41	105.58	Q9ZRA4	AB19A	sub3
*Eucgr.C03536*	1079.85	930.58	667.89	227.95	439.95	6177.00	Q9ZRA4	AB19A	sub3
*Eucgr.D00606*	58.97	124.94	334.92	267.52	238.79	182.00	Q05349	12KD	sub4
*Eucgr.I01276*	3.24	3.83	1.87	7.70	10.80	26.74	Q6NMM0	SAU61	sub4
*Eucgr.F03208*	38.00	1.29	0.67	2.78	0.74	0.95	Q9SQ80	G2OX1	sub6
*Eucgr.F04125*	22.90	32.45	8.65	10.03	4.30	3.94	P46687	GASA3	sub3
*Eucgr.K02472*	20.46	20.90	18.74	117.00	41.92	58.02	Q6NMQ7	GASA6	sub5
*Eucgr.F00192*	13.57	17.63	27.34	38.65	23.74	14.97	Q8LC30	RAP21	sub1
*Eucgr.F02317*	1.08	0.47	1.55	58.00	1.66	0.87	O22174	ERF08	sub1
*Eucgr.F02691*	0.04	0.36	23.00	34.00	0.00	0.19	Q70II3	EF110	sub1
*Eucgr.H01659*	0.02	0.05	0.53	1.38	0.00	0.00	Q9SZ06	EF109	sub1
*Eucgr.K00128*	19.27	9.67	16.3	75.95	18.5	13.04	Q9LY05	EF106	sub1
*Eucgr.F04203*	0.13	0.20	8.69	4.30	0.27	0.15	Q9FGF8	ABR1	sub4
*Eucgr.G01970*	0.15	0.37	3.51	6.09	1.74	0.32	Q9LYU3	EF113	sub4
*Eucgr.H03965*	5.20	12.24	20.35	18.47	21.78	6.23	P42736	RAP23	sub4
*Eucgr.C04221*	30.5	24.82	14.00	22.97	11.44	29.80	Q9XI33	WIN1	sub6
*Eucgr.F02319*	7.73	5.56	7.53	12.26	6.01	2.54	Q8LC30	RAP21	sub6
*Eucgr.I00422*	12.92	11.51	3.99	14.41	20.31	13.61	O65665	ERF60	sub6
*Eucgr.K00126*	69.17	62.26	47.43	89.37	59.17	42.78	Q8VY90	EF105	sub6
*Eucgr.A01146*	18.41	11.39	6.04	13.68	12.62	5.29	Q8L8B8	LOG3	sub6
*Eucgr.B02321*	5.67	7.47	35.5	11.99	13.25	6.79	O81077	ABAH2	sub1
*Eucgr.C01524*	7.03	4.49	2.78	23.39	14.27	12.84	Q9SKK0	EBF1	sub1
*Eucgr.C03157*	33.47	38.36	13.15	25.79	30.28	12.39	Q9FUJ1	CKX7	sub6
*Eucgr.E01149*	13.82	13.43	23.66	19.39	9.18	6.47	Q949P1	ABAH1	sub1
*Eucgr.G01437*	0.04	0.29	1.74	6.04	1.81	1.43	Q9LJK2	ABAH4	sub1
*Eucgr.G03093*	3.61	1.72	1.54	3.81	0.47	0.53	Q8S8E3	PYL6	sub6
*Eucgr.I01127*	15.24	21.06	9.75	27.58	26.97	8.78	Q8W3P8	AOG	sub1
*Eucgr.I01201*	0.14	0.61	0.11	0.42	3.41	3.39	Q6RYA0	SABP2	sub4
*Eucgr.J00169*	6.76	4.55	2.08	5.09	1.23	0.54	O80920	PYL4	sub6
*Eucgr.K02472*	20.46	20.9	18.74	11.17	41.92	58.02	Q6NMQ7	GASA6	sub5
*Eucgr.B03374*	1.38	2.09	4.49	1.70	1.41	0.96	Q9ZWS9	ARR3	sub1
*Eucgr.B02620*	11.64	26.09	57.13	113.34	90.20	95.91	Q39182	DEF02	sub2
*Eucgr.H05052*	223.79	163.36	302.39	893.65	3003.64	3268.99	Q07502	DEF	sub4

**Table 5:** Differentially expressed genes encoding transcription factors in different groups.

Transcription Factors	G1	G2	G3	AS	BS
C_2_H_2_			1		1
CPP					1
ERF	7			1	2
MYB	4		3	1	2
NAC	6			1	1
RAV					1
WRKY				2	5
B3				3	
bHLH	3		1	1	
bZIP				2	
C_3_H	3			2	
Dof				2	
G2-like				3	
HD-ZIP	1			1	
HSF	1	1		2	
MYB_related	1		3	2	
NF-YA				1	
TCP				1	
MIKC_MADS			1		
LBD	1				
SBP	1				

**Table 6:** Differentially expressed transcription factors in different groups.

Groups of DEGs	Gene ID	Gene Names	T1	T2	CK	log_2_FPKM (T1/CK)	*p* Value	log_2_FPKM (T2/CK)	*p* Value
AT	*Eucgr.H00996*	WRKY23	1.57	4.82	8.19	−2.38	0.00	−0.76	0.18
AT	*Eucgr.B03520*	WRKY75	6.93	9.67	24.10	−1.80	0.00	−1.32	0.03
BT	*Eucgr.B04010*	WRKY26	15.39	8.77	4.79	1.68	0.00	0.87	0.07
BT	*Eucgr.K02940*	WRKY33	8.01	5.62	2.84	1.50	0.00	0.99	0.06
BT	*Eucgr.C00675*	WRKY50	26.21	17.53	7.47	1.81	0.00	1.23	0.02
BT	*Eucgr.E04011*	WRKY6	4.21	2.48	1.07	1.98	0.00	1.22	0.08
BT	*Eucgr.I01633*	WRKY75	1.72	4.70	0.54	1.66	0.16	3.11	0.01

## Data Availability

The data obtained and used in this study are available from the corresponding author upon reasonable request. The data are not publicly available due to privacy.

## Supplementary Materials

The following supporting information can be downloaded at: https://www.mdpi.com/article/10.3390/genes15010060/s1, Figure S1: Leaves at different treatments of ZQUA44 and ZQUB15; Table S1: Primers for qRT-PCR validation.
