# Supplementary figures and images for: Comparative Transcriptome Analysis of High- and Low-Growth Genotypes of Eucalyptus urophylla in Response to Long-Term Nitrogen Deficiency
Source: Genes (Basel). 2023 Dec 30;15(1):60. doi: 10.3390/genes15010060 (PMC10815775; doi:10.3390/genes15010060)

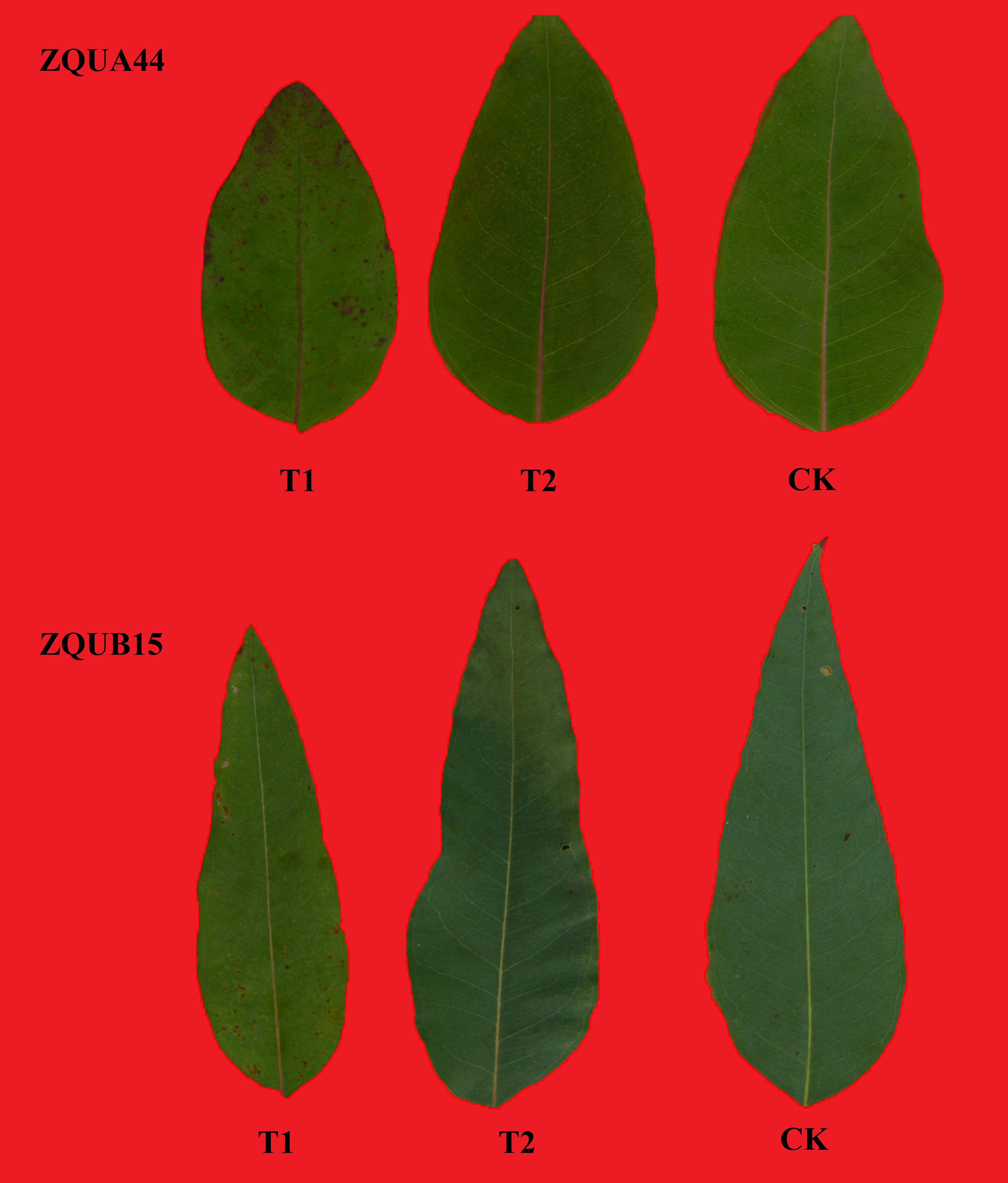

Supplement: Supplementary file 1 [file genes-15-00060-s001.zip › Fig.S1.tif]
